# Job preference of preventive medicine students during the COVID-19 pandemic: a discrete choice experiment survey in Shandong Province, China

**DOI:** 10.1186/s12909-023-04873-2

**Published:** 2023-11-21

**Authors:** Zhuang Tian, Wei Guo, Min Zhai, Hongmin Li

**Affiliations:** 1 School of Public Health, Jining Medical University, Jining, 272067 China; 2 Public Health Service Center in Rencheng District, Jining, 272412 China

**Keywords:** Job preference, Preventive medicine students, Rural health services, Discrete choice experiment

## Abstract

**Background:**

Public health workers are a crucial part of the health workforce, particularly during the coronavirus disease (COVID-19) pandemic. They play an important role in achieving universal health coverage and sustainable development goals. Human resources in public health in China are in short supply, their distribution is unequal, and their turnover rate is high. A discrete choice experiment (DCE) was applied to investigate preventive medicine students’ preferred job choice criteria and trends in trade-offs by calculating the marginal rate of substitution between these criteria. This study identified the properties of jobs primarily selected by preventive medicine students and estimated the monetary value of each attribute.

**Methods:**

Based on discussions and in-depth interviews with preventive medicine students and a literature review, we developed a DCE that assessed how students’ stated preferences for a certain choice were influenced by several job attributes, including location, salary, *bianzhi*, career development opportunities, working environment, and workload. We applied this DCE to preventive medicine students in Shandong Province, China, using a brief, structured questionnaire. Conditional logit models were used to estimate the utility of each job’s attributes. Willingness to pay (WTP) was estimated as the ratio of the value of the coefficient of interest to the negative value of the cost attribute.

**Results:**

A total of 307 respondents completed the questionnaire, and 261 passed the internal consistency test. All the attributes were statistically significant. Career development opportunities and work locations were the most important factors for the respondents. Preference heterogeneity existed among respondents, e.g., 3-year medical education college students placed a higher value on jobs with *bianzhi* compared to 5-year medical education college students. Furthermore, rural students’ WTP for a job located in the county or city is much lower than that of urban students.

**Conclusions:**

The heterogeneity of attributes indicates the complexity of job preferences. Monetary and nonmonetary job characteristics significantly influenced the job preferences of preventive medicine students in China. A more effective policy intervention to attract graduates to work in rural areas should consider both job incentives and the backgrounds of preventive medicine graduates.

## Background

The International Year of Health and Care Workers (YHCW) has been proposed for 2021 to honor their commitment to combating the COVID-19 pandemic [1]. Public healthcare workforce has an inalienable and non-negligible need to battle new infectious diseases and other public health threats [2]. A close association exists between the concentration of qualified public health workers and key health outcomes in developing countries [3]. The attainment of sustainable development goals (SDGs) and universal health coverage (UHC) is essentially hampered by this gap [4].

However, global concerns regarding the shortage and misdistribution of a qualified public health workforce exist [5], which affect almost all countries, particularly those that are relatively poor [6, 7]. The tendency of public health physicians in China to visit urban areas causes a shortage in rural areas, leading to insufficient health coverage and poor health conditions among rural people [8, 9]. For example, the number of health technicians per thousand people in 2019 was 11.1% in urban areas and 4.96% in rural areas. In most areas, fewer than two people exist in township health centers for every 1,000 rural population, although the two-week prevalence rate in rural areas (32.2%) was significantly higher than that in urban areas (23.2%) in 2018 [10]. This highly uneven distribution between urban and rural areas is entrenched because cities offer better incomes (e.g., the potential for private practice), more opportunities for career progression, better infrastructure, and more social amenities than rural areas. Simultaneously, the human resource turnover rate increased in township hospitals because of the imbalance between recruitment conditions and job attraction, incentive mechanism construction, and career development [11].

The shortage of health workers in rural China, coupled with severe losses in public health, calls for coordinated context-sensitive actions to build a health workforce of sufficient quantity and quality. The lack of high-quality and practical talent is the most fundamental factor restricting the development of grassroots healthcare. Therefore, it has attracted attention from governments and researchers regarding finding solutions that attract more health workers to rural areas.

As a medical specialty, preventive medicine teaches students about clinical medicine as well as public health and is unique in combining direct patient care clinical skills and public health expertise [12]. Therefore, preventive medical students will be the most important future source of healthcare workers. However, a mismatch between the requirements and the annual production of preventive medicine students has been observed in China. Appropriate supply of the health workforce is a fundamental problem. Additionally, the recruitment and retention of public health workers are causing problems [13], particularly in rural China.

Many DCE studies have been conducted for medical students' job preferences from different majors [14–17] in China and many other countries [18, 19]. Valuable information was provided about the economic and non-economic factors influencing the job choices of the medical students. But evidence on the job priorities and preferences of public health students [20] was limited especially in regular higher education institutions (HEIs). Therefore, this study explores the most relevant job attributes that preventive medicine students in HEIs would prefer to have while working in rural areas of China using the discrete choice experiment (DCE) technique to help policymakers devise recruitment and retention strategies in rural areas.

## Methods

### Setting and sample

This study was conducted in Shandong Province, which has a population of approximately 100.7 million, of which the urban and rural populations account for 61.51% and 38.49% [10]. In 2019, the gross regional product of Shandong Province amounted to CNY 7106 billion, ranking it as the third largest economy in China. However, the number of staff in township health centers per thousand rural people in Shandong Province is 1.64, ranking 12th in China [21].

Preventive medicine is often treated as a specialty of medicine, and thus clinical training is regarded as the foundation for additional training in public health [22]. Public health education is mostly an undergraduate education, five years after high school, unlike the postgraduate public health degrees in the USA [23]. Public health education programs in China's higher education institutions (HEIs) can be divided into four categories based on educational attainment levels: three-year junior college (Da Zhuan), four- or five-year undergraduate/bachelor, three-year master, and three-year PhD programs [24]. Considering that the type of higher education institution may be related to their choice to work in rural areas [25], two 3-year junior colleges (Shandong Medical College and Heze Medical College) and one 5-year medical education university (Jining Medical university) were included in this study. Preventive medicine students in their final year of study were selected because they were likely to have a preliminary or better understanding of their future career plans.

Based on the simple sampling strategy proposed by Orme [26], the minimum number of respondents required for this study was calculated by *N* > 500c/(t*a). 500 is a fixed variable; *c* is the highest number of levels in any attribute; *t* represents the number of choice experiment questions for each DCE questionnaire; *a* refers to the number of choices contained in each discrete choice experiment question. This equals 500*3/(9*2)≈84.

### Selection of attributes and levels

The development of attributes and levels is a critical step in DCEs [27]. A literature review and qualitative studies were conducted to ensure that the attributes and levels included were most meaningful for the respondents.

First, we identified 13 attributes from a literature review: salary, working environment, work location, *bianzhi,* workload, career development, training opportunities, management style, welfare, children’s education opportunities, transportation, residents’ recognition and respect, and organizational culture [28–30].

These attributes should be identified as the main factors considered by preventive medicine students’ when making career decisions. Therefore, we confirmed the significance of attributes and attribute levels through interviews conducted with six students majoring in preventive medicine. Five public health professors conducted a final review of the attributes and attribute levels deduced through a literature review and student interviews. The usefulness of management style, training opportunities, residents’ recognition and respect, organizational culture, and unfeasibility of welfare, children’s education opportunities, and transportation were excluded from the analysis, and six attributes, namely location, salary, *bianzhi*, career development opportunities, working environment, and workload, were selected. Attribute levels were determined based on literature review findings and the levels currently being applied to public health agencies. Table 1 presents the six attributes included in the final design.

**Table 1:** DCE attributes and attribute levels

Attributes	Levels	*Explanation*
**Location**	Township or village County City	Refers to work in different level public health agency at different regions. Public health agency includes Health administration agencies at all levels, disease control agencies, health supervision agencies, maternal and child health agencies, chronic disease prevention and treatment agencies, community health service agencies, etc
**Salary**	CNY 2000 CNY 5000 CNY 8000	Monthly income which represents the pre-tax salary
***Bianzhi***	No Yes	Refers to established posts which means more stable
**Career development opportunities**	Insufficient Some Sufficient	Represents the opportunities of getting promoted
**Working environment**	Poor Normal Excellent	Refers to the physical and social environment about the work
**Workload**	Heavy Medium Light	Includes the workload in the daytime and the conditions of working overtime

### Discrete choice experiment design

In total, 3^5^ × 2 = 486 hypothetical job scenarios will be produced through full factorial design, then a total of (486 × 485)/2 choice sets will be generated, and those are not feasible for a single respondent to choose. Orthogonal designs were used to reduce the full set of scenarios to a more manageable level of 18 choice sets using a D-efficient design with *Ngene* DCE design software. *Ngene s*oftware was also used to divide the 18 choice sets into two versions to avoid overloading the participants. One of the choice sets in each version was included twice as a consistency test; however, data from the repeated choice sets was not included in the final analysis. All participants were randomized to receive one of the two versions of the DCE questionnaire.

### Data collection

The survey was conducted online through *Wenjuanxing* (https://www.wjx.cn/) in December 2020. It was the largest online survey company in the country. Selected students completed a questionnaire. Electronic data collection, which provides a fast and cost-effective method to collect data, is increasingly being used in DCE. Although previous studies have indicated that using an online method to collect data may lead to certain forms of interviewer bias, there is no indication that online surveys yield results inferior to paper-based surveys [31]. Thus, this study adopted a web-based survey, considering that all students currently have access to the Internet.

The DCE questionnaires were explained in detailed on the cover letter. The questionnaire consisted of two sections. The first section included general characteristics of participants. The second section contained 10 choice sets of hypothetical job scenarios. The third set of hypothetical job scenarios in each version of the questionnaire was repeatedly included as the 10th set of hypothetical job scenarios to test the respondents' understanding of the discrete selection experiment questions and control the quality of the research.

### Data analysis

According to the principle of regression model selection, the smaller the value of the Akaike Information Criterion or Bayesian Information Criterion, the more accurate and reasonable the corresponding regression model in the context of this research. Therefore, this study selected the conditional logit model for regression analysis.

The “price” attribute was specified as a continuous variable to facilitate the calculation of willingness to pay (WTP), which is the monetary value that people place on different attributes of the job scenarios.

We also conducted a simulation study to predict the uptake rates and understand the extent to which the probability of choosing a given post changes as the levels of the attributes change. This finding will be useful for policymakers.

The logit probability of choosing alternative i rather than alternative j is given by:

$${P}_{i}=\frac{EXP({V}_{i})}{\sum_{j=1}^{N}EXP({V}_{j})}$$

## Results

A total of 307 preventive medicine students completed the questionnaires and were recruited for the survey. There were 177 participants from Jining Medical University, 83 from Shandong Medical College and 47 from Heze Medical College. Participants who provided inconsistent answers to the repeated-choice sets were excluded from the main analysis (*n* = 46, 14.98%), and the detailed results reported below were based on the remaining 261 preventive medicine students.

### General characteristics

No significant differences existed between the participants who passed or failed the consistency test. The results indicated that females (63.8%) were the majority. Most students (56.7%) came from townships or villages; 198 (64.5%) had brothers or sisters; 46.9% of the participants spent *CNY* 800–1500 per month; and 51.8% of the participants indicated their annual family income was less than *CNY* 50,000. Respondents’ general characteristics are presented in Table 2.

**Table 2:** General characteristics for preventive medicine students from 3 medical universities/colleges, n (%)

**Characteristics**	**All (*n* = 307)**	**Participants who passed the consistency test (*n* = 261)**	**Participants who failed the consistency test (*n * = 46)**	**χ**^**2**^	***P* value**
**Sex**				0.207	0.649
**Male**	111(36.2)	93(35.6)	18(39.1)		
**Female**	196(63.8)	168(64.4)	28(60.9)		
**School**				3.374	0.185
**Jining medical university**	177(57.7)	145(55.6)	32(69.6)		
**Shandong medical college**	83(27.0)	75(28.7)	8(17.4)		
**Heze medical college**	47(15.3)	41(15.7)	6(13.0)		
**Hometown**				2.862	0.239
**City**	71(23.1)	56(21.5)	15(32.6)		
**County**	62(20.2)	53(20.3)	9(19.6)		
**Township or village**	174(56.7)	152(58.2)	22(47.8)		
**Only child**				2.433	0.119
**Yes**	109(35.5)	88(33.7)	21(45.7)		
**No**	198(64.5)	173(66.3)	25(54.3)		
**Consumption level (CNY per month)**				3.206	0.201
**0-**	53(17.3)	43(16.5)	10(21.7)		
**800-**	144(46.9)	128(49.0)	16(34.8)		
**1500-**	110(35.8)	90(34.5)	20(43.5)		
**Family income(CNY per year)**				5.182	0.269
**0-**	88(28.7)	80(30.7)	8(17.4)		
**30,000-**	71(23.1)	57(21.8)	14(30.4)		
**50,000-**	50(16.3)	41(15.7)	9(19.6)		
**70,000-**	40(13.0)	32(12.3)	8(17.4)		
**90,000-**	58(18.9)	51(19.5)	7(15.2)		

### Job preferences among preventive medicine students

The main results of the conditional logit model are presented in Table 3. As expected, the mean coefficients of all the attributes were statistically significant. Among the nonmonetary attributes, preventive medicine students expressed the highest stated preference for a job with sufficient career development (*β* = *1.085,P* = 0.000), followed by a city location (*β* = *0.972*, *P* = 0.000). Although workload had a positive effect on respondents (*β* = *0.243*, *P* = 0.002), it did not appear to be as important as the other attributes.

**Table 3:** Main effects model results and estimated willingness to pay (WTP) for job attributes among preventive medicine students (based on clogit estimates; *n* = 261)

Attribute levels	β	SE	*P* value	*WTP*(*CNY*)	95%CI	
**Bianzhi(ref:no)**	0.435	0.053	0.000	1364.1	1035.3	1692.9
**Location(ref: Township or village)**						
**County**	0.803	0.088	0.000	2518.5	1975.7	3061.3
**City**	0.972	0.079	0.000	3047.9	2561.3	3534.5
**Career development(ref:insufficient)**						
**Some**	0.612	0.087	0.000	1918.4	1380.9	2455.9
**Sufficient**	1.085	0.084	0.000	3403.4	2886.7	3920.1
**Working environment(ref:Poor)**						
**Normal**	0.208	0.131	0.112	652.6	-152.4	1457.6
**Excellent**	0.428	0.123	0.001	1344.0	585.4	2102.6
**Workload(ref: Heavy)**						
**Medium**	0.192	0.083	0.02	603.0	95.7	1110.2
**Light**	0.243	0.077	0.002	762.3	289.6	1235.0
**Monthly income**	0.000319	0.0000148				
**LR chi2(11)**	1031.63					
**Number of obs**	5,220					
** Log likelihood**	-1293.2988					
**AIC**	2608.598					
**BIC**	2680.76					

*WTP* Willingness to pay, *CNY* Chinese yuan, *SE* Standard error, *95% CI* 95% confidence intervals, *AIC* Akaike information criterion, *BIC* Bayesian information criterion

### Estimated WTP for job attributes

The WTP results are also presented in Table 3 and are used for a relative comparison. Respondents were willing to pay CNY 3403.4 in monthly income for a job with sufficient career development rather than for a job with insufficient career development. They assigned a value of CNY 3047.9 per month to a work location in the city compared to work in a township or village. Regarding workload, the students were willing to pay only CNY 762.3 to get a job with a light workload as compared to a job with a heavy workload.

### Subgroup analysis

Table 4 presents the results of the subgroup analyses. Evidently, 3-year medical education students valued *bianzhi* at CNY 2105.7, whereas 5-year medical education students valued bianzhi at CNY 757.5. For the former, the workload and working environment became insignificant, whereas 5-year medical education students were significantly willing to pay more to work with a light workload and better working environment.

**Table 4:** Results of clogit models by different subgroups

**Attribute**	**3-year medical education colleges (*n* = 11**2**)**				**5-year medical education university(*n* = 149)**			
	β	*SE*	*P*	*WTP(CNY)*	β	*SE*	*P*	*WTP(CNY)*
***Bianzhi* (ref:no)**	0.720	0.086	0.000	2105.7	0.234	0.070	0.001	757.5
**Location(ref: Township or village)**								
**County**	0.889	0.142	0.000	2600.6	0.759	0.114	0.000	2458.0
City	1.012	0.126	0.000	2960.0	0.950	0.103	0.000	3075.9
**Career development (ref: insufficient)**								
**Some**	0.706	0.140	0.000	2063.8	0.559	0.114	0.000	1812.2
Sufficient	1.192	0.139	0.000	3487.6	1.051	0.108	0.000	3406.1
**Working environment (ref: Poor)**								
**Normal**	0.248	0.216	0.251	724.8	0.223	0.167	0.182	721.7
Excellent	0.265	0.201	0.188	775.0	0.534	0.160	0.001	1731.4
**Workload(ref: Heavy)**								
**Medium**	0.049	0.130	0.706	143.7	0.286	0.110	0.009	925.3
Light	0.152	0.120	0.205	445.0	0.304	0.102	0.003	984.8
Monthly income	0.000342	0.0000234	0.000		0.000309	0.0000196	0.000	
**Attribute**	**Urban(*n* = 109)**				**Rural(*n* = 152)**			
	β	*SE*	*P*	*WTP(CNY)*	β	*SE*	*P*	*WTP(CNY)*
***Bianzhi* (ref:no)**	0.353	0.083	0.000	1211.1	0.531	0.073	0.000	1522.1
**Location(ref: Township or village)**								
**County**	0.999	0.132	0.000	3427.9	0.643	0.123	0.000	1844.2
City	1.269	0.120	0.000	4353.6	0.736	0.108	0.000	2110.0
**Career development (ref: insufficient)**								
**Some**	0.520	0.137	0.000	1785.3	0.724	0.117	0.000	2075.4
Sufficient	1.008	0.122	0.000	3459.1	1.171	0.120	0.000	3358.2
**Working environment (ref: Poor)**								
**Normal**	0.268	0.201	0.183	919.4	0.181	0.177	0.307	519.0
Excellent	0.257	0.181	0.155	881.7	0.620	0.176	0.000	1779.2
**Workload(ref: Heavy)**								
**Medium**	0.196	0.122	0.107	673.8	0.160	0.117	0.170	459.8
Light	0.189	0.116	0.104	649.6	0.270	0.104	0.010	774.8
Monthly income	0.000291	0.0000224	0.000		0.000349	0.0000204	0.000	
**Attribute**	**Male(*n* = 93)**				**Female(*n* = 168)**			
	β	*SE*	*P*	*WTP(CNY)*	β	*SE*	*P*	*WTP(CNY)*
***Bianzhi* (ref:no)**	0.362	0.087	0.000	1161.8	0.476	0.068	0.000	1444.5
**Location(ref: Township or village)**								
**County**	0.799	0.135	0.000	2564.5	0.825	0.119	0.000	2506.6
City	0.995	0.128	0.000	3196.7	0.967	0.102	0.000	2936.8
**Career development (ref: insufficient)**								
**Some**	0.498	0.142	0.000	1600.8	0.689	0.112	0.000	2093.6
Sufficient	0.801	0.127	0.000	2571.6	1.283	0.115	0.000	3896.9
**Working environment (ref: Poor)**								
**Normal**	0.127	0.211	0.547	408.3	0.289	0.169	0.088	876.8
Excellent	0.446	0.199	0.025	1433.5	0.408	0.159	0.010	1240.2
**Workload(ref: Heavy)**								
**Medium**	0.096	0.130	0.458	309.6	0.233	0.109	0.032	709.1
Light	0.044	0.121	0.720	139.8	0.373	0.101	0.000	1132.3
Monthly income	0.000311	0.0000235	0.000		0.000329	0.0000197	0.000	

Among all the attributes of the jobs, rural students’ WTP for a job located in the county or city is much lower than that of urban students, namely students from a county or city valued a city location with a WTP of CNY 4353.6 compared to students who came from townships or villages with a WTP of CNY 2110.0. Rural students valued career development more, whereas urban students focused on workplace location.

Apart from workload which appeared to be insignificant for males, and career development were valued more by female students, the preferences for other job attributes between males and females were relatively similar.

**Fig. 1:**
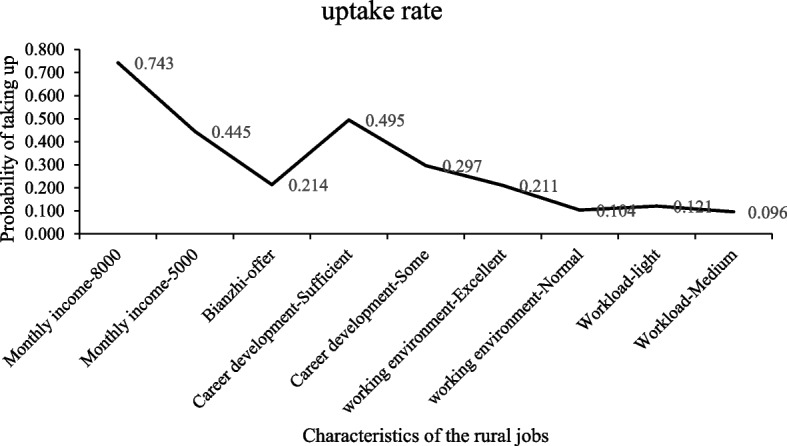
Policy simulation demonstrating changes in probabilities as the rural jobs changes. Baseline job posting: monthly income of *CNY* 2000, no bianzhi, insufficient career development, poor work environment and heavy workload)

We used the coefficients from the conditional logit model to transform the data into percentages of preventive medicine students estimated to take a rural job compared to an urban job with various incentives provided, as presented in Fig. 1. For single incentives, increasing monthly income from 2,000 to 8,000 CNY had the largest effect on preference for rural postings. With improved career development from insufficient to sufficient, 49.5% of the respondents were expected to select a rural job.

## Discussion

This study elicited preferences for job attributes among preventive medicine students at vocational colleges and application-oriented university using the DCE. All six attributes (including economic and non-economic factors) significantly affected students’ job choices.

Our findings confirmed that monthly income had a significant impact on the job choices of preventive medicine students, which was consistent with the results of other studies [15, 32]. Earlier studies in China also shows that the first pressure source for public health workers was low income [33] and salary level was regarded as the most direct cause of the loss of public health institutions [34].

For single incentives, increasing monthly income from 2,000 to 8,000 CNY had the largest effect on preference for rural postings. A study in rural Zambia identified salary top-ups for health workers in rural areas as the most effective incentive [35]. However, financial incentives are not the only considerations when preventive medicine students make job-choice decisions.

The two most important non-economic job attributes for preventive medicine students were career development and location. Regarding career development, many students voiced concerns about being forgotten in rural posts when it came to promotion and career development opportunities [36]. A qualitative study by Wang [37] in China revealed that in rural areas, the training of grassroots health personnel is insufficient and does not match their job requirements, which is not conducive to improving their professional skills. Concerns about slow promotions and lack of mentoring or career development were among the most frequently mentioned concerns by physicians in rural districts [38, 39]. Therefore, enriching career development opportunities should be a priority when attempting to improve the retention of health workers in rural China [40, 41].

Work location is another important nonmonetary factor. A literature review of middle- and low-income countries also showed that poor working conditions and a lack of equipment or infrastructure contribute to the flight of healthcare personnel from rural areas [42]. Additionally, living conditions in most rural areas remain poor compared to those in urban areas concerning convenience of living, transportation, and children’s education resources, which could be another main barrier to health manpower retention in rural areas. The subgroup analysis suggested substantial heterogeneity among preventive medicine students regarding the value of work location. Compared to students from rural areas, those from urban areas showed a much stronger preference for working in cities rather than in counties or rural areas. This follows what has been reported for rural Liberia [43] where exposure to rural areas is associated with a much higher willingness to work in rural areas. Therefore, attracting and retaining students from rural backgrounds in rural areas is a more feasible and cost-effective strategy. The Chinese government adopted a plan to strengthen the primary healthcare workforce by increasing the enrollment of students from rural areas and providing free medical education, called rural-oriented medical education (RTME) [44]. The RTME program aims to enroll medical students, primarily from rural areas, to work in township hospitals for six years after graduation. However, a study in Shaanxi, China, indicated that only 1.3% of RTMS intended to remain after a six-year contract that worked for 6 years in a township hospital expired [45]. Many health-worker recruitment and retention strategies implemented in rural Zambia also appear to have little or no impact on maintaining health workers in rural areas [35]. To be effective, interventions must be implemented in bundles, combining different packages of interventions according to the variety of factors influencing health workers’ decisions to work in rural or remote areas. Therefore, the design and implementation of these programs should be improved, which requires multi-sectoral efforts except in the education department. Human resources and social security departments should introduce policies that favor preventive medicine students in primary health institutions concerning *bianzhi*, title promotion, appointments, etc. Financial departments must provide salary subsidies and performance wages. Health departments must provide more systematic and standardized training opportunities.

A 3-year medical education primarily trains doctors at the grassroots level [46]; however, the subgroup analysis revealed little difference between the 3-year medical students and 5-year medical students for the value of work location, which results in a large gap in public health talent, especially in urban grassroots and rural areas. This reveals that the level of public health education is unclear [47], the training of public health talents does not match the needs of society, which leads to structural unemployment [48], and the social recognition of public health majors is low [49]. It concludes with a call for action for the medical education system to adapt to meet the needs of public healthcare learners during COVID-19 and even beyond [23, 50]. Educational authorities and higher medical schools must closely integrate changes in social needs and optimize the structure of talent training to achieve an effective connection between talent supply and the demands of public health [24, 51, 52].

Regarding the working environment, providing an excellent working environment is moderately effective, which is consistent with a study conducted in China [53]among Ph. D. students majoring in public health but differs from what has been reported in Uganda [54] and Indonesia [32] among nursing students. Compared to other medical students, preventive medicine students show little consideration for a better working environment, possibly because information construction in the healthcare system brings new ways of working during COVID-19 [55]. During the “Thirteenth Five-Year Plan” period, China’s grassroots health informatization developed rapidly, which manifested itself in the rapid popularization of information systems [56], enhanced interconnection, and rich and diverse business applications [57].With the COVID-19 pandemic, we have witnessed a further major shift in which several digital technologies have gained prominence [58]. Digital transformation requires a new understanding of the concepts of public health and universal health coverage (UHC), which reflects the extent to which digital technologies offer new tools through which public health goals can be achieved [59].

*Bianzhi* was found to have a relatively minor effect, which was consistent with another study in medical students in China [17], but contrary to other Chinese studies emphasizing that *Bianzhi* is crucial in the construction of health personnel [60, 61]. The Chinese government has implemented policies to strengthen the grassroots through the reform of its health system, which has stressed the issue of salary distribution and treatment guarantees for rural doctors. Many provinces in China have launched specific measures to guarantee that the income level at the grassroots level, such as primary medical staff implement equal pay for equal work regardless of *Bianzhi* [62]. Therefore, *bianzhi* is not attractive for presenting preventive medicine students in the context of deepening the reform of the health system.

Workload was valued as the least important attribute, which is consistent with what has been reported by Vujicica et al. [63], particularly for the 3-year medical students and those who are male, workload appears to be insignificant. But contrary to previous studies with nursing students in China [15], which found that working strength were the most important non-economic job attribute. This may be related to the fact that compared to public health personnel, nurses are required to work night shift with a heavier workload which can be certificated from complaints about fatigue and fatigue related illnesses in shift nurses [64, 65]. And the stereotype of hard-working Chinese has been around for a long time, Chinese students grow up with different values, including a different estimation of the importance of working hard. However, when they are officially working, focusing on stress management to avoid job burnout becomes necessary [66]. A study related to job preferences conducted by Chinese medical staff in the post-pandemic era showed that workload is a key factor in medical staff’s job choices [67].

This study has some limitations. First, because a difference exists between revealed and stated preferences, our study analyzes the stated preferences of the students for hypothetical scenarios, which may not necessarily reflect the choices they would make in a real setting. Second, the five key attributes in the design may not fully reflect respondents’ decisions in the real world because of the complexity of job decision-making. Finally, because the representativeness of the sample in the study was limited, caution is required when generalizing the findings of this study to all preventive medicine students in China.

## Conclusions

This study analyzed the major attributes that influence the job preferences of 261 preventive medicine students with a DCE and calculated the WTP for improvements over each attribute using the coefficients of the attributes obtained in the regression analysis. We found that salary, work location, career development, working environment, workload, and *bianzhi* were the attributes that influenced job choice. This study confirmed that monetary attributes and nonmonetary attributes significantly influenced preventive medicine students’ job preferences. Apart from raising monthly income to a certain level, prioritizing strategies that can supply sufficient career development may be more effective in China. Moreover, preference heterogeneity exists in preventive medicine students’ job preferences, which should also be considered when developing more effective policy incentive packages.

## Data Availability

The datasets used and/or analyzed in the current study are available from the corresponding author upon reasonable request.

## Abbreviations

DCE: Discrete choice experiment
AIC: Akaike information criterion
BIC: Bayesian information criterion
SE: Standard error
WTP: Willingness-to-pay
